# Exploration of the Determinants of Subjective Health and Depression Using Korean Longitudinal Study of Aging Data

**DOI:** 10.3390/healthcare12141424

**Published:** 2024-07-17

**Authors:** Kyung-A Sun, Joonho Moon

**Affiliations:** 1 Department of Tourism Management, Gachon University, Sungnam-si 13120, Republic of Korea; kasun@gachon.ac.kr; 2 Department of Tourism Administration, Kangwon National University, Chuncheon 24341, Republic of Korea

**Keywords:** elderly, subjective health, depression, medical expenses, eating-out expenses, regular exercise

## Abstract

Aging is an imperative issue in Korean society, and a healthy life is important for a better quality of life for older adults. Therefore, the purpose of this research was to investigate the determinants of subjective health and depression in middle-aged and elderly Korean individuals. This study used three attributes as the determinants of subjective health and depression, including the curve linear effect of medical expenses and eating-out expenses and the linear impact of regular exercise. We utilized the Korean Longitudinal Study of Aging (KLOSA) to determine the associations between five attributes: subjective health, depression, medical expenses, eating-out expenses, and regular exercise. Research panel data were employed as the data source. The study period was between 2018 and 2020. This research implemented various multiple linear panel regression econometric analysis instruments: ordinary least squares, random effects, and fixed effects. The mean age of survey participants was 72.10 years, and 35 percent of participants were female. The number of observations for data analysis was 7197. The results revealed that medical and eating-out expenses had a curved linear effect on subjective health and depression. Moreover, regular exercise positively affected subjective health and resulted in less depression. These findings may inform policy decisions that promote regular exercise and manage medical and eating-out expenses, thereby enhancing subjective health and mitigating depression.

## 1. Introduction

According to the Korean Statistical Information Service [1], the proportion of the population over 65 years of age was 17.5 percent (approximately 8.9 million) in 2022. Moreover, the Korean Statistical Information Service [1] reported that the number of people over 65 years was approximately 9.4 million in 2023; the increase in the older adult population was approximately 0.5 million within one year. Additionally, the Korean Statistical Information Service [1] projected that the proportion of older adults will reach approximately 30 percent in 2035. Moreover, the extant literature states that aging causes various problems, such as reduced productivity, pension problems, local extinction crises, digital literacy problems, alienation, isolation, etc. [2,3,4]. Such facts indicate that Korean society is aging, and the Korean government might need to prepare for problems caused by aging. As a baseline for preparation, the first step might be to understand the characteristics of older adults. Given this necessity, this research aimed to understand the behavioral characteristics of middle-aged and elderly Korean individuals and provide information for governmental policy design.

The first area of this research was subjective health. Many studies have examined subjective health, because a healthy physical and mental condition is important for everybody [5,6,7]. Scholars have also contended that a healthy condition is essential for a better life [8,9]. The extant literature documents that subjective health is affected by various attributes [10,11]. A large body of the extant literature indicates that subjective health is worthy of further investigation. In addition, numerous scholars have explored subjective health by using this attribute as the explained variable [9,12,13]. Moreover, Moon et al. [14] documented that subjective health is a precondition for a better quality of life for aging individuals. They stated that subjective health is crucial for inspecting the behavioral characteristics of the elderly. Therefore, this research used subjective health as the first dependent variable.

Another main attribute of this work is depression. Depression is a negative mental state that causes undesirable effects, such as insomnia and suicide [15,16,17]. Many studies have been conducted on depression, due to its seriousness [18,19,20], and researchers have shown that depression is caused by diverse factors [20,21]. Sivertsen et al. [22] also claimed that quality of life is negatively affected by depression in the case of older people. In fact, Statistics Korea [23] also claimed that elderly suicide in Korean society has been one of the main causes of death among this population. Scholars have stated that depression could become a reason for suicide [21,22]. Thus, depression is worth investigating, because finding causes could be the starting point for therapy in the case of older adults.

Consumption patterns could become cues for understanding individual behavioral characteristics, because people spend their money according to their values [23,24]. Furthermore, Statistics Korea [25] reported that middle-aged and old Korean individuals desire to work after retirement because of living expenses and are interested in the pension system in their old age. This implies that financial conditions related to consumption patterns could become the main focus of research on middle-aged and old Korean individuals. Hence, this research examined spending patterns and explored two attributes: medical and eating-out expenditures. Among elderly individuals, medical expenditures become more crucial because the likelihood of illness is higher than among younger individuals [26,27,28,29]. Indeed, Statistics Korea [25] reports that the medical expenditure of older Korean people is increasing, suggesting that medical expenses could become a burden. Moreover, Khan and Mahumud [30] noted that it is controversial whether medical services are necessities or luxuries. This study answers this question by examining the impact of medical expenses.

Next, this research assessed the impact of eating-out costs. Scholars have contended that eating out enables individuals to consume flavorsome food and engage in an enjoyable experience [31,32,33]; however, excessive eating out is harmful to health, because the probability of obesity increases as the frequency of eating out increases [34,35,36]. Moreover, diminished marginal utility could be applied to eating-out costs because repeated consumption decreases the marginal utility of eating out [37,38]. Statistics Korea [25] also stated that the elderly Korean population prefers food consumption at home because of financial burdens. It can be inferred that middle-aged and older Korean individuals are somewhat conservative regarding eating-out costs. This work thus elucidated the effects of eating out on older Korean adults.

The next area of this work is regular exercise. Taylor [39] stated that regular exercise is an essential element for promoting a healthy condition. Prior research noted that regular exercise is indispensable for a healthier life, because exercise lowers individuals’ likelihood of illness [40,41,42], which could be applicable to elderly individuals. In addition, Zhang et al. [43] documented that physical exercise plays a significant role in the treatment of depression, and Callow et al. [44] claimed that physical activity is an important attribute for the better mental health of older adults. Furthermore, investigations into the association between regular exercise and subjective health could be worthwhile, because a reversal of causality could occur. Thus, this research investigated the effect of regular exercise on subjective health and depression. Based on the argument of prior studies, this research focused on determining whether regular exercise affects the health of older Korean adults. Helliwell [45] documented that health, economic, and social factors are the pillars of better living. By following their framework, this research selected three attributes as explanatory variables. In detail, both economic and health factors could contribute to medical expenses, and eating-out expenses could comprise both economic and social factors. Moreover, regular exercise belongs to the health area.

All things considered, the goal of this research was to examine the determinants of subjective health and depression, with a focus on Korean older adults. The determinants were medical expenses, eating-out expenses, and regular exercise. To be specific, the aims of this study were to test the curve linear effect of medical and eating-out costs on both depression and subjective health. Another goal of this research was to examine the impact of regular exercise on subjective health and depression in older Korean adults. The attributes’ directions were likely to appear in opposition to one another because subjective health and depression exhibit positive and negative statuses, respectively. The following are the research hypotheses proposed in this study:

**Hypothesis 1:**  *Medical expenses exert a U-shaped effect on subjective health.*

**Hypothesis 2:**  *Medical expenses exert an inverted U-shaped effect on depression.*

**Hypothesis 3:**  *Eating-out expenses exert an inverted U-shaped effect on subjective health.*

**Hypothesis 4:**  *Eating-out expenses exert a U-shaped effect on depression.*

**Hypothesis 5:**  *Regular exercise exerts a positive effect on subjective health.*

**Hypothesis 6:**  *Regular exercise exerts a negative effect on depression.*

In order to test the research hypotheses, this research adopted longitudinal archival data consisting of multiple time points and participants. Using these data, this research employed econometric instruments for data analyses. Moreover, this study ensured the reverse effect of attributes, because subjective health and depression could become positive and negative factors, respectively. By carrying this out, this research sheds light on the literature by clarifying the effect of medical and eating-out expenses on both the subjective health and depression of Korean elderly individuals. Additionally, this research contributes to the literature by disclosing the impact of regular exercise on subjective health and depression. Such elucidation might add to the literature, because the topic of medical and eating-out expenses is controversial [30,33,34]. In addition, the outcome of this research could have policy implications for realizing better welfare among older adults. Namely, the outcomes of this study could provide guidelines for allocating government budgets for medical, food, and physical activities relative to older Koreans.

## 2. Review of the Literature and Hypothesis Development

### 2.1. Subjective Health

The extant literature defines subjective health as a self-appraised health condition [7,8]. Prior studies have also documented subjective health results from assessments that consider physiological and psychological factors [9,11]. Numerous prior studies chose subjective health as the focal point. For example, Sun et al. [8] examined the Chinese population’s characteristics using subjective health as the main element. Paakkari et al. [46] also explored the subjective health of school-aged children. Von Steinbuechel et al. [47] assessed subjective health in patients with brain injuries. Additionally, Gustainienė and Endriulaitienė [6] studied subjective health in sales managers. Gaitán-Rossi et al. [13] examined the determinants of subjective health in migrants. Moreover, Poortinga et al. [9] observed the significant and positive impact of green space on subjective health. Låftman et al. [12] researched gender differences using subjective health in the school domain. In addition, numerous scholars have scrutinized the characteristics of subjective health in older adults [3,7,11,48]. In detail, Heo and Choi [3] explored the impact of physical health on subjective health by employing multiple regression as the main instrument and using Korean older adults as the study subject. Wei et al. [48] researched Chinese individuals as research targets using subjective health as the dependent variable and multiple regression for data analysis. The review of the literature revealed that subjective health is widely scrutinized in various domains.

### 2.2. Depression

Depression refers to a mental condition associated with deep sadness and a gloomy mood [18,19,20]. Scholars have contended that depression is a mental illness that sometimes causes undesirable effects, such as alcoholism, insomnia, and suicide [15,16,17]. Numerous studies have been conducted on depression, due to the aforementioned severe effects. In detail, Othman et al. [49] documented the determinants of depression in university students. Breidenbach et al. [50] explored the antecedents of depression in breast cancer survivors. Additionally, Smith and White [51] performed a systematic review to identify the characteristics of depression. In addition, Sahni et al. [52] revealed the influential attributes of depression among Indian individuals. Similarly, Admemola et al. [53] inspected explanatory variables of depression in Ghana and Nigeria. Many studies have addressed the characteristics of depression in elderly individuals [54,55,56]. Shahar et al. [55] performed interviews with Malaysian older adults to investigate the characteristics of depression. Zenebe et al. [56] performed a systematic literature review using older adults as study subjects. Cénat et al. [57] researched Canadian cases using depression as the dependent variable. Wegbom et al. [58] scrutinized the determinants of depression by examining pregnant women. In a similar vein, Li et al. [59] explored older Chinese adults to observe the antecedents of depression. This literature review thus indicates that depression has been frequently examined across various fields and in numerous populations, including elderly individuals. Furthermore, Helliwell [45] contended that better subjective health conditions are likely to be realized with respect to the following three dimensions: economic, health, and social dimension. Considering such an argument, this research scrutinizes the determinants of health conditions: economic, health, and social attributes.

### 2.3. Medical Expenses

Medical expenses refer to the costs associated with health conditions [60,61] and are categorized as both prevention and treatment costs [27,62]. Prevention comprises care that maintains a healthy condition, whereas treatment comprises care that enables recovery from illness [26,63]. In general, the extant literature states that treatment is regarded as a necessity, while prevention could be regarded as a necessity or a luxury [27,64]. Scholars have argued that elderly individuals tend to spend more money on medical costs because the likelihood of illness is higher than that in younger individuals [28,29]. Hence, previous studies addressed the fact that the consumption of medical services is likely to become a fixed cost for elderly individuals [28,65]. However, prior studies suggested that medical services sometimes become luxury goods, depending on the individual’s financial condition [63,64]. Some older adults pay for luxury services to improve their health; thus, scholars contended that healthcare services can either be a necessity or a luxury [64,66]. In the necessity case, medical expenses are likely to negatively impact subjective health because the treatment process has negative mental and physical consequences. In contrast, the extant literature reports that medical expenses can enhance psychological and physiological conditions when they are regarded as luxury services—for example, spas, luxury medical services in hospitals, and luxury healthcare services [66,67,68]. Therefore, medical expenditure for luxury goods is anticipated to improve individuals’ subjective health and mental condition. Thus, a curved linear effect with respect to medical expenditure can be anticipated, because the consumption patterns and financial condition of middle-aged and elderly people could vary, rendering a medical service either a necessity or a luxury. Therefore, this research study proposes Hypotheses 1 and 2.

### 2.4. Eating-Out Expenses

Eating-out expenses are costs associated with eating outside the house [69,70]. Eating out refreshes an individual’s emotional and physical condition, because people become energized when consuming new foods and interacting socially with others [31,32,33]. Scholars have also argued that eating out allows individuals to experience new atmospheres [31,71]. Thus, eating out likely promotes mental health. In contrast, other studies have addressed the drawbacks of eating out: obesity, high blood pressure, and cost burden [34,35,36]. Additionally, scholars have claimed that goods consumption is related to diminishing marginal utility, because the marginal utility is reduced in the repetitive consumption of goods and services with respect to individual spending [37,38,72]. Li and Hsee [59] also claimed that excessive spending results in regret on the individual’s part, because their utility declines through unnecessary consumption. Das et al. [73] demonstrated declined marginal utility by examining consumers of wine and solar energy. This could be applied to eating out because the utility decreases over time as negative outcomes increase, which include risks of obesity, high blood pressure, and heart attacks. Hence, a curved linear effect of eating-out costs should be reasonably assumed when considering the argument of diminished marginal utility. We thus propose Hypotheses 3 and 4.

### 2.5. Regular Exercise

Prior studies have indicated that regular exercise plays a significant role in improving health conditions and reducing the probability of disease, because exercise enhances the metabolic system and increases positive hormone secretion [40,74]. Martinsen [75] contended that exercise can treat depression. Lindwall et al. [76] also demonstrated a negative association between regular exercise and depression in an elderly Swedish population. Similarly, De Moor et al. [41] found a negative effect of regular exercise on depression. Moreover, Heo and Choi [3] researched elderly Korean individuals, and the findings implied that exercise plays an essential role in promoting subjective health conditions. Kanamori et al. [42] also revealed a positive relationship between subjective health and regular exercise by analyzing archival data using multivariable logistic regression. Moreover, Lim and Hyun [77] documented that regular exercise (e.g., Pilates and yoga) is a crucial attribute that promotes subjective health by analyzing data using regression analysis. In a similar vein, George and Reddy [78] implemented a systematic literature review, and their findings indicated that regular exercise plays a significant role in improving elderly mental health condition. Osali [79] executed an experimental study, and the results disclosed that older adults’ health condition—analyzed with respect to blood samples—was enhanced by regular exercise. In summary, the extant literature reported that regular exercise plays an essential role in improving individual health condition. This observation could be applied to middle-aged and older people. This study thus proposes Hypotheses 5 and 6.

## 3. Methods

### 3.1. Research Model and Design

**Figure 1:**
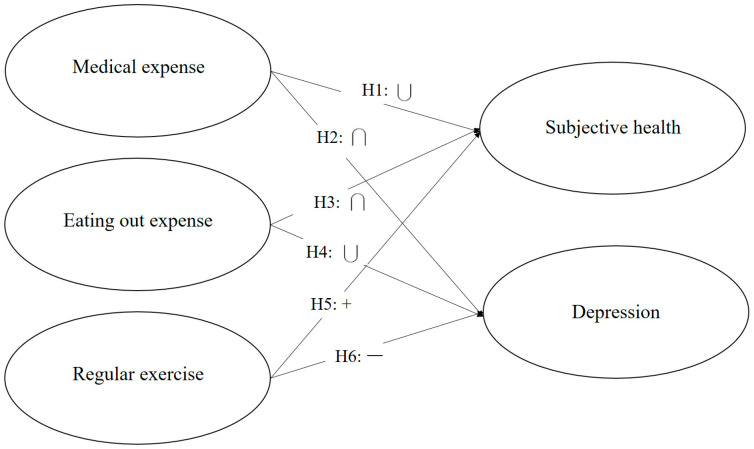
Research model.

Figure 1 shows the research model of this study. Subjective health and depression are dependent variables, and medical expenses, eating-out expenses, and regular exercise are independent variables. Medical expenses demonstrate a U-shaped relationship with subjective health, while medical expenses show an inverted U-shaped relationship with depression. In contrast, eating-out expenses demonstrate an inverted U-shaped impact on subjective health and a U-shaped impact on depression. Finally, regular exercise positively affects subjective health and negatively impacts depression.

### 3.2. Data Collection

This research used Korean Longitudinal Study of Aging (KLOSA) research panel data and was conducted between 2018 and 2020. The Korean Employment Information Service provides the KLOSA dataset to the public every two years, and the aim of KLOSA is to understand the behavioral characteristics of the elderly as comprising basic information for policy making. The investigation was implemented longitudinally, which followed up with the survey’s participants. KLOSA research panel data have often been used in quantitative research to determine the behavioral characteristics of middle-aged and elderly Korean individuals [80,81,82]. It can be inferred that the data quality is suitable for reliable statistical inferences. KLOSA research panel data provide senior citizen information obtained from a survey and cover multiple periods and participants [83,84]. Because 2018 and 2020 are the years with the most recently available information, this research study selected the 2018 and 2020 periods. The data comprised an unbalanced panel: survey participants were not fully matched in both periods (2018: N = 3607; 2020: N = 3590). Therefore, the total number of individuals observed in this study was 7197 (N = 7197).

### 3.3. Description of Variables

Table 1 displays the measurement of variables. Subjective health (SHE) was measured using a five-point scale (1 = very poor; 5 = very good) using the following statement: How do you evaluate your own health status? The measurement of DEP used a four-point scale (1 = rarely; 4 = always) with the following statement: For last week, how frequently did you experience depression? Table 1 also depicts the measurements of monthly medical expenses (MDEs) and monthly eating-out expenses (EOE), where the unit is KRW 10,000 (Korean Won). Additionally, regular exercise (RGE) (0 = no; 1 = yes) and sex (SEX) (0 = male; 1 = female) were binary variables. Age (AGE) was measured using the physical age of survey participants. Personal asset (PAS) measurement is the value of the assets possessed by the survey’s participants (unit: KRW 10,000). Finally, this study used COVID-19 (COV) as a dummy variable (0 = 2018; 1 = 2020).

### 3.4. Data Analysis

For statistical analysis, this study used STATA 13. This study analyzed descriptive statistics by computing the mean, standard deviation (SD), minimum, maximum, skewness, and kurtosis. Skewness refers to the asymmetric degree of the data, while kurtosis is the thickness degree of the tail [83,84,85]. Additionally, a correlation matrix analysis was conducted to inspect the relationship between variables. For research hypothesis testing, the current study implemented three econometric instruments: ordinary least squares (OLS), fixed effects (FEs), and random effects (REs) [83,84,85]. Panel data analysis minimized the bias in the estimation. OLS attempt to minimize the errors in the estimation. FE aim to minimize the omitted variable’s panel data bias using dummy variables to control the time effect [83,84,85]. In the FE model, COV was used as the attribute for the year effect. In addition, RE refers to the econometric model that incorporates unobserved effects into the model [83,84]. The use of the three econometric models ensured result consistency in terms of significance and direction [83,84]. This research also excluded participant effects from the regression model, because these can seriously sacrifice the degree of freedom with respect to estimations. This study also computed the variation inflation factor to detect multicollinearity in estimations using ten as the threshold [84]. Furthermore, this study implemented quadratic regression model analysis, including squared variables [84,85]. MDE^2^ and EOE^2^ were used in the regression model. After estimating coefficients in a quadratic regression model, differentiation was conducted to reach the first-order condition, enabling the calculation of the point for either minimizing or maximizing the value of dependent variables [84,85]. The following is the research model of this study:

*SHE_it_* = *β*_0_ + *β*_1_*MDE_it_* + *β*_2_*MDE*^2^*_it_* + *β*_3_*EOE_it_* + *β*_4_*EOE*^2^*_it_* + *β*_5_*RGE_it_* + *β*_6_*SEX_it_* + *β*_7_*AGE_it_* + *β*_8_*PAS_it_* + *β*_9_*COV* + *ε*

*DEP* = *β*_0_ + *β*_1_*MDE_it_* + *β*_2_*MDE*^2^*_it_* + *β*_3_*EOE_it_* + *β*_4_*EOE*^2^*_it_* + *β*_5_*RGE_it_* + *β*_6_*SEX_it_* + *β*_7_*AGE_it_* + *β*_8_*PAS_it_* + *β*_9_*COV* + *ε*

where *ε* is the residual, *i* denotes the *i*th participants, and *t* denotes the *t*th time.

## 4. Results

### 4.1. Descriptive Statistics and Correlation Matrix

Table 2 shows the descriptive statistics. The mean value of SHE was 2.90, and the standard deviation was 0.85. The mean value of DEP was 1.48; the standard deviation was 0.72. The descriptive information also included MDE (mean = 10.26, SD = 18.90, minimum = 0, and maximum = 1000) and EOE (mean = 9.49, SD = 10.54, minimum = 0, and maximum = 150). Table 2 also presents the mean values of the following dummy variables: RGE (mean = 0.35), SEX (mean = 0.35), and COV (mean = 0.35). The mean value of AGE was 72.10, and the standard deviation was 9.19. The participants’ ages ranged from 57 to 102. Additionally, the mean value of PAS was 30,935.33, with a standard deviation of 42,081.95. All kurtosis and skewness values were 0, with the exception of COV (kurtosis = 0.86).

Table 3 describes the correlation matrix. SHE negatively correlated with DEP (r = −0.314, *p* < 0.05), MDE (r = −0.078, *p* < 0.05), and AGE (r = −0.397, *p* < 0.05), while SHE positively correlated with EOE (r = 0.122, *p* < 0.05), RGE (r = 0.097, *p* < 0.05), SEX (r = 0.104, *p* < 0.05), and PAS (r = 0.105, *p* < 0.05). DEP positively correlated with MDE (r = 0.072, *p* < 0.05) and AGE (r = 0.179, *p* < 0.05), whereas it negatively correlated with EOE (r = −0.064, *p* < 0.05), RGE (r = −0.067, *p* < 0.05), SEX (r = −0.031, *p* < 0.05), and PAS (r = −0.046, *p* < 0.05). MDE positively correlated with EOE (r = 0.125, *p* < 0.05) and PAS (r = 0.101, *p* < 0.05); EOE positively correlated with PAS (r = 0.341, *p* < 0.05). RGE positively correlated with SEX (r = 0.041, *p* < 0.05) and PAS (r = 0.160, *p* < 0.05); however, RGE negatively correlated with AGE (r = −0.198, *p* < 0.05).

### 4.2. Results of Hypothesis Testing

Table 4 illustrates the results of multiple regression analyses using SHE as the dependent variable. All three econometric models, OLS, RE, and FE, were statistically significant (*p* < 0.05). The VIF values implied that the estimation was less likely undermined by multicollinearity. SHE was significantly influenced by MDE (β = −0.007, *p* < 0.05) and MDE^2^ (β = 8.32 × 10^−6^, *p* < 0.05), and the point computed by the first-order condition for the minimization of SHE by MDE was 741.18. Additionally, SHE was significantly determined with respect to EOE (β = 0.006, *p* < 0.05) and EOE^2^ (β = −9.10 × 10^−5^, *p* < 0.05), and the point computed by the first-order condition for maximization of SHE via EOE was 44.22. Moreover, RGE exerted a positive effect on SHE (β = 0.094, *p* < 0.05). SEX (β = 0.156, *p* < 0.05) and PAS (β = 1.35 × 10^−6^, *p* < 0.05) were positively associated with SHE; AGE was negatively associated with SHE (β = −0.034, *p* < 0.05).

Table 5 shows the results of hypothesis testing using DEP as the dependent variable. All three econometric models, including OLS, RE, and FE, were statistically significant (*p* < 0.05). The VIF values indicate that the estimation was less likely biased with respect to multicollinearity. DEP was significantly impacted by MDE (β = 0.004, *p* < 0.05) and MDE^2^ (β = −2.78 × 10^−6^, *p* < 0.05), and the point calculated via the first-order condition for the maximization of DEP by MDE was 736.23. Additionally, SHE was significantly influenced by EOE (β = −0.003, *p* < 0.05) and EOE^2^ (β = 3.91 × 10^−5^, *p* < 0.1), and the point computed using the first-order condition for the minimization of DEP by EOE was 43.03. In addition, RGE negatively impacted DEP (β = −0.074, *p* < 0.05). SEX (β = −0.034, *p* < 0.1) and PAS (β = −421 × 10^−7^, *p* < 0.05) were negatively related to SHE, and AGE was positively related to DEP (β = 0.012, *p* < 0.05).

## 5. Discussion

This study examined the impacts of medical and eating-out expenses and regular exercise on the subjective health and depression of senior Korean citizens. It was revealed that medical expenses exerted a U-shaped and inverted U-shaped impact on subjective health and depression, respectively. This suggested that medical services could be categorized as luxury goods for certain older adults who can afford higher expenses. Moreover, the results showed that eating-out expenses exhibited an inverted U-shape relationship with respect to subjective health, while a U-shaped relationship between eating-out expenses and depression was shown. It can be inferred that an excessive eating-out budget is damaging to the health condition of the elderly because eating out too frequently might result in poor health conditions, such as high blood pressure, obesity, and serious depression [34,35,36,86]. Compared to the mean values of medical and eating-out expenses, the computed optimal point was substantially lower. This indicated that most middle- and old-aged individuals possessed insufficient budgets for medical and eating-out services. Next, the findings of this study revealed the positive impact of regular exercise on better subjective health conditions. That is, regular exercise improves health conditions and decreases the level of depression in the elderly Korean population. The findings of this study are aligned with the results of previous research in the case of Dutch [41] and Japanese [42] populations. Indeed, Palumbo et al. [87] reported that regular exercise is critical for the better health condition of individuals after conducting a systematic literature review. The findings of this study could be used as evidence to support such an argument. Regarding sex, the results suggested that females were mentally and physically healthier than males. It is possible that female individuals were less likely to be isolated than older male adults because there were fewer variances in the case of women compared to men. To be specific, older male adults tended to be retired, while there were fewer female retirement cases with respect to old age. Namely, older male adults are more likely to face hardship with respect to how they spend their time after retirement, which is a radical change in life condition. However, older female adults are less likely to face this phenomenon in old age, which might be an explanation for the significance of sex in terms of depression and subjective health. Additionally, the results uncovered that older survey participants were less healthy. In addition, the results documented that wealthy older adults had a healthier mental and physical condition. Finally, the results reported that the subjective health of older adults was worse during COVID-19.

## 6. Conclusions

### 6.1. Theoretical Implications

This study first provides a theoretical contribution to the literature by clarifying the curved linear effect of medical and eating-out expenses on subjective health and depression. This is because prior studies have rarely explored the impact of medical and eating-out expenditure on depression and subjective health with respect to linear curve impacts. Because this research study addressed this research gap, the results have theoretical value. Hence, the results shed light on the literature by demonstrating that medical expenses could be construed as a necessity or luxury with respect to the impact on both subjective health and depression. Moreover, this study potentially fills this research gap by confirming the explanatory power of diminished marginal utility theory in the case of older Korean people with respect to the effect of eating-out expenses on both subjective health and depression [38,72,88]. Next, this research study computed both maximization and minimization points for subjective health and depression via the first-order condition. Given the outcome of the computation, this research study observed, via comparisons with the mean value, that senior citizens in Korean society did not have sufficient resources for medical and eating-out expenses. In addition, this research study externally validated the findings of prior studies by uncovering the positive effect of regular exercise on both subjective health and depression [39,74,75]. In addition, this study contributes to the literature by supporting the causal effect of regular exercise on health condition, because reverse causality could be anticipated.

### 6.2. Policy Implication

This study provides policy implications. First, policymakers need to focus more on minimizing the burden of medical expenses in order to promote the health of elderly individuals. Compared to the estimated value from the quadratic regression model, the mean medical expenditure value was quite small, implying that the budget for medical expenses is insufficient. Therefore, policymakers need to carefully invest in medical care for older individuals, which is associated with health insurance plans. Moreover, the results revealed that eating-out budgets among elderly individuals were insufficient with respect to improving subjective health and lowering depression. Government policies could take into account methods for supporting eating-out expenses for middle-aged and elderly individuals in order to improve their health condition. This could be accomplished in two ways: by offering direct financial support for elderly individuals and by providing subsidies for restaurant business managers. Additionally, policymakers might need to consider the allocation of resources in order to help elderly individuals exercise. This could be accomplished by not only allotting more resources to senior welfare centers for equipment and exercise programs but also supporting private exercise centers for middle-aged and elderly individuals. Such efforts could promote the mental and physical health of such individuals. Furthermore, senior welfare policymakers should concentrate more on male senior citizens because their health condition is worse than female individuals. Moreover, government policies need to focus more on the older and poorer elderly population because their health might be worse.

### 6.3. Future Lines of Research

This study has some limitations. First, this study’s research sample was limited to only middle-aged and elderly Korean individuals. Future studies could select different samples, such as global or youth populations. Such efforts could render the results of this study more generalizable. Moreover, the explanatory power of depression in this research study was only approximately four percent, indicating that a stronger predictor of depression may exist. Therefore, it could be valuable for future studies to investigate the determinants of depression using more attributes. In addition, future research might be able to use more refined measurement items because most items in this study depended on a single measurement item. Such efforts could provide increased understanding with respect to middle-aged and old individuals.

## Figures and Tables

**Table 1:** Measurement of variables.

Variable	Measurement
Subjective health (SHE)	1 = very poor; 5 = very good
Depression (DEP)	1 = rarely; 4 = always
Medical expenses (MDE)	Monthly medical expenses (unit: KRW 10,000)
Eating-out expenses (EOE)	Monthly eating-out expenses (unit: KRW 10,000)
Regular exercise (RGE)	0 = no; 1 = yes (once a week)
Sex (SEX)	0 = male; 1 = female
Age (AGE)	Physical age of survey participants
Personal assets (PAS)	Personal assets (KRW 10,000)
COVID-19 (COV)	0 = 2018; 1 = 2020

**Table 2:** Descriptive statistics (N = 7197).

Variable	Mean	SD	Minimum	Maximum	Skewness	Kurtosis
SHE	2.9	0.85	1	5	0	0
DEP	1.48	0.72	1	4	0	0
MDE	10.26	18.9	0	1000	0	0
EOE	9.49	10.54	0	150	0	0
RGE	0.35	0.47	0	1	0	0
SEX	0.35	0.47	0	1	0	-
AGE	72.1	9.19	57	102	0	0
PAS	30,935.33	42,081.95	0	818,000	0	0
COV	0.49	0.49	0	1	0.86	-

Note: SD: standard deviation; SHE: subjective health; DEP: depression; MDE: medical expenses (unit: KRW 10,000); EOE: eating-out expenses (unit: KRW 10,000); RGE: regular exercise; SEX: sex; AGE: age; PAS: personal assets (unit: KRW 10,000); COV: COVID-19.

**Table 3:** Results of the correlation matrix.

Variable	1	2	3	4	5	6	7
1. SHE	1						
2. DEP	−0.0314 *	1					
3. MDE	−0.078 *	0.072 *	1				
4. EOE	0.122 *	−0.064 *	0.125 *	1			
5. RGE	0.097 *	−0.067 *	0.011	0.183 *	1		
6. SEX	0.104 *	−0.031 *	0.023 *	0.070 *	0.041 *	1	
7. AGE	−0.397 *	0.179 *	0.018	−0.198 *	−0.061 *	−0.030 *	1
8. PAS	0.105 *	−0.046 *	0.101 *	0.0341 *	0.160 *	0.048 *	−0.085 *

Note: * *p* < 0.05; SHE: subjective health; DEP: depression; MDE: medical expenses (unit: 10,000 KRW); EOE: eating-out expenses (unit: KRW 10,000); RGE: regular exercise; SEX: sex; AGE: age; PAS: personal assets (unit: KRW 10,000).

**Table 4:** Results of the subjective health hypothesis test case.

Variable	Model1 OLS β (*t*-Stat)	Model2 RE β (Wald)	Model3 FE β (*t*-Stat)	VIF
Intercept	5.314 (68.58) **	5.314 (68.58) **	5.314 (68.56) **	
MDE	−0.007 (−10.85) **	−0.007 (−10.85) **	−0.007 (−10.85) **	2.19
MDE^2^	8.32 × 10^−6^ (7.53) **	8.32 × 10^−6^ (7.53) **	8.32 × 10^−6^ (7.53) **	2.15
EOE	0.006 (3.72) **	0.006 (3.72) **	0.006 (3.72) **	3.56
EOE^2^	−9.10 × 10^−5^ (−3.23) **	−9.10 × 10^−5^ (−3.23) **	−9.10 × 10^−5^ (−3.23) **	3.2
RGE	0.094 (4.88) **	0.094 (4.89) **	0.094 (4.88) **	1.05
SEX	0.156 (8.09) **	0.156 (8.23) **	0.156 (8.09) **	1.01
AGE	−0.034 (−34.05) **	−0.034 (−34.29) **	−0.034 (−34.05) **	1.06
PAS	1.35 × 10^−6^ (5.83) **	1.35 × 10^−6^ (5.84) **	1.35 × 10^−6^ (5.83) **	1.16
COV	-	-	0.001 (0.05)	
F-value	210.58 **	-	187.15 **	
Wald χ^2^	-	1684.61 **	-	
R^2^	0.1908	0.1623	0.1908	

Note: SHE is the dependent variable; OLS denotes ordinary least squares; RE is random effect; FE is the fixed effect; VIF stands for the variation inflation factor; ** *p* < 0.05. Point by first-order condition for SHE: −Δ/ΔMDE = 741.18, −Δ/ΔEOE = 44.22. SHE: subjective health; MDE: medical expenses (unit: KRW 10,000); EOE: eating-out expenses (unit: KRW 10,000); RGE: regular exercise; SEX: sex; AGE: age; PAS: personal assets (unit: KRW 10,000); COV: COVID-19.

**Table 5:** Results of the depression hypothesis test case.

Variable	Model4 OLS β (*t*-Stat)	Model5 RE β (Wald)	Model6 FE β (*t*-Stat)	VIF
Intercept	0.589 (8.01) **	0.589 (8.18) **	0.585 (7.95) **	
MDE	0.004 (4.88) **	0.004 (6.24) **	0.004 (4.87) **	2.19
MDE^2^	−2.78 × 10^−6^ (−3.24) **	−2.78 × 10^−6^ (−2.70) **	−2.79 × 10^−6^ (−3.26) **	2.15
EOE	−0.003 (−2.39) **	−0.003 (−2.30) **	−0.003 (−2.37) **	3.56
EOE^2^	3.91 × 10^−5^ (1.77) *	3.91 × 10^−5^ (1.49)	3.98 × 10^−5^ (1.80) *	3.2
RGE	−0.074 (−4.20) **	−0.074 (−4.12) **	−0.071 (−4.03) **	1.05
SEX	−0.034 (−1.92) *	−0.034 (−1.93) *	−0.026 (1.44)	1.01
AGE	0.012 (13.16) **	0.012 (13.74) **	0.013 (13.28) **	1.06
PAS	−4.21 × 10^−7^ (−2.22) **	−4.21 × 10^−7^ (−1.96) *	−4.15 × 10^−7^ (−2.18) **	1.16
COV	-	-	−0.044 (−2.55) **	
F-value	39.41 **	-	35.14 **	
Wald χ^2^	-	319.63 **	-	
R^2^	0.0428	0.0428	0.0437	

Note: DEP is the dependent variable; OLS denotes ordinary least squares; RE denotes random effect; FE is fixed effect; VIF stands for the variation inflation factor; * *p* < 0.1, ** *p* < 0.05. Point by first-order condition for SHE: −Δ/ΔMDE = 736.23, −Δ/ΔEOE = 43.03. DEP: Depression; MDE: medical expenses (unit: KRW 10,000); EOE: eating-out expenses (unit: KRW 10,000); RGE: regular exercise; SEX: sex; AGE: age; PAS: personal assets (unit: KRW 10,000); COV: COVID-19.

## Data Availability

The original data presented in the study are publicly available on the Korean Employment Information Service’s website: https://survey.keis.or.kr/ (accessed on 11 April 2022).
